# Big Five Personality Profiles in the Norwegian Special Operations Forces

**DOI:** 10.3389/fpsyg.2020.00747

**Published:** 2020-05-05

**Authors:** Tom Hilding Skoglund, Thor-Håvard Brekke, Frank Brundtland Steder, Ole Boe

**Affiliations:** ^1^Faculty of Health Sciences, UiT The Arctic University of Norway, Tromsø, Norway; ^2^Psychology Department, Bjørknes College, Oslo, Norway; ^3^Norwegian Armed Forces, Oslo, Norway; ^4^Norwegian Defence Research Establishment, Oslo, Norway; ^5^University of South-Eastern Norway, School of Business, Drammen, Norway

**Keywords:** military, SOF, special forces, Big Five, personality

## Abstract

This study is the first to report on Big Five personality traits of employees in the Norwegian military Special Operations Forces (NORSOF). Three research questions were formulated for this study, aiming to investigate (1) whether age, number of combat-deployments and rank (OR/OF) had an impact on the personalities of NORSOF employees, (2) possible personality differences between personnel organized in the underlying departments of the NORSOF, and (3) if there were personality differences between SOF-operators and conventional forces applicants. SOF-operators from the Norwegian Special Operations Commando (FSK) and the Norwegian Naval Special Operations Commando (MJK) constituted 40% of the total NORSOF sample (*N* = 190), whilst the term SOF-support categorized the larger proportion of non-operators. Results indicated that younger employees tended to be lower on emotional stability than older colleagues, and that those without any combat-deployments were somewhat higher on agreeableness and a bit lower on emotional stability relative to employees with such experience. Additionally, personnel with officer ranks (OF) were higher on extraversion compared to specialists (OR). Results did not show any significant intradepartmental differences in mean personality trait scores. Compared to male applicants for basic officer training in conventional forces (*N* = 662), SOF-operators (all males) were less extroverted, less agreeable, and slightly more emotionally stable. The authors conclude that the NORSOF attracts and recruits personnel with similarities in their Big Five personalities. Furthermore, we suggest that the personality profile that emerged for the “average” Norwegian SOF-operator is a functional one, especially when considering the desired future image of the Special Forces operative as a Warrior-Diplomat.

## Introduction

Special Operations Forces (SOFs) are typically characterized by stringent personnel selection systems, advanced military training regimes and versatile mission forms, including asymmetrical warfare, making such units one the most demanding of all military specialties. Such units have a wider range of capabilities and often operate more independently, usually in smaller teams, relative to regular military forces. Having a flexible structure, SOFs can function as liaisons between military forces, civilian organizations and different law enforcement agencies in international operations – in addition to being a tactical level asset for special missions. Personnel requirements such as versatility, agility, effectiveness, speed and surprise, working both independently and in direct support to others, are often highlighted in published materials concerning SOFs (NATO, 2012). To distinguish a SOF-operator from soldiers in other military units, Spulak (2007) used the term “Elite warrior” in order to pinpoint that operators typically demonstrate superior task performance relative to the performance of the larger military population, thus underlining the “specialness” of SOFs. The term “Warrior-Diplomat” has been used to describe the ideal future image of SOF-personnel (Berg-Knutsen and Roberts, 2015), whereby operators are capable of combining tactical level competency with insights in societal perspectives, such as political and cultural processes, thus enabling optimal job performance across sectors and institutions in an international context.

More detailed job analyses from SOFs and specific psychological requirements for its personnel are, however, confidential information in most countries. This clouds the possibility of gaining more precise knowledge of the psychological characteristics of employees, especially for its “tip of the spear” operators. Published empirical studies with samples from SOFs are scant. Thus, studies with such samples, acknowledging security concerns such as personnel anonymity, are needed in the military psychology literature. Empirical studies can contribute to improvements in selection systems and other human resource strategies within these important military assets, and ultimately support the SOF truth that “people are more important than hardware” (USSOCOM, 2019, p. 57).

The Norwegian Special Operations Forces (NORSOF) are organized with two Special Forces and a joint staff. The Norwegian Special Operations Commando (NORSOC or FSK in Norwegian) is a formerly Army affiliated department, whereas the Norwegian Naval Special Operations Commando (NORNAVSOC or MJK in Norwegian) is a formerly Navy affiliated department. The former has its main historical roots in paratrooper operations, the latter mainly in frogman operations (for a historical perspective, see Olsen and Thormodsen, 2014). Both the FSK and the MJK are on national and international standby for special operations and counter-terrorism actions, and both units have frequent deployments to conflict areas abroad, where the capacity building of security forces in other states has been one of the main official missions (Hedenstrom and Kristiansen, 2016).

The operator selection to the FSK and the MJK are separate, and both selection systems are considered extremely demanding regarding physical and psychological requirements. Thus, only a few candidates complete the selection phase and become trained as combat-ready operators. In addition to the operators, the NORSOF has different personnel categories, collectively named “SOF-support” for the purpose of the present study. This category has personnel with different military specialties and backgrounds, and with the common characteristic that they have not been through an operator selection process, either in the FSK or the MJK, and therefore are not fully trained nor approved as SOF-operators. SOF-support personnel contribute to the operational capacity of the FSK and the MJK in many ways, for example by participating in training for operations, by providing contributions in diverse missions and combat-deployments, or by executing leadership from different hierarchies in the organization.

By measuring the psychological characteristics of personnel in the NORSOF, this study aims to investigate possible intradepartmental differences in individual attributes, and examine if the carefully selected and highly trained operators have psychological dissimilarities relative to those in other military personnel categories. The present study aims to examine these questions by means of Big Five personality testing. The Big Five framework is essentially equivalent to the Five Factor Model (FFM) in the concepts used for personality descriptions (John et al., 2008). The Big Five (or FFM) is a system for describing the phenomenon of personality differences from a lexical standpoint, a method for conceptualizing personality dating back to at least Allport and Odbert’s (1936) classical work of extracting 18,000 person-describing words from the English dictionary. During the 1980s, the Big Five framework, and measurement methods thereof, emerged as a cemented discipline within personality psychology (Digman, 1990; Scroggins et al., 2008), spawning voluminous research publications (see for example Widiger, 2017). While the NORSOF has used Big Five testing for internal educational and organizational purposes, published Big Five personality studies of personnel employed by the NORSOF are to the authors’ knowledge non-existent.

The present study used a Big Five test recently developed by the Norwegian Armed Forces, designed to measure factors with the same meaning as the definitions seen in the widespread NEO-PI-R/3 test (Costa and McCrae, 1992; McCrae et al., 2005; McCrae and Costa, 2010): Neuroticism, emotional instability rather than adjustment; Extraversion, represented by a need to be outgoing and active as opposed to being an introvert; Openness to experience, being imaginative and having broad interests as opposed to being traditional and down to earth; Agreeableness, a compassionate style toward others rather than being antagonistic; Conscientiousness, a tendency to be well organized and goal-oriented as opposed to an easy-going- and non-directional style. Big Five testing (and diverse personality tests with resemblance to the Big Five) have been widespread in military contexts, especially in the domain of personnel selection (Salgado, 1998; Campbell and Knapp(eds), 2001; Stark et al., 2014). Personality traits are often considered relevant for selection decisions in both military and civilian contexts, as they (especially conscientiousness) tend to add incremental validity after controlling for general mental ability (Schmidt and Hunter, 1998; Darr, 2009).

The Big Five traits are viewed as stable characteristics, disposing for individual patterns of cognitions, emotions and behaviors (McCrae and Costa, 2010). Through adult years, trait level changes have, however, been demonstrated. Changes seem to predominate in the age span 20–40, for example by increasement of emotional stability (Roberts and Mroczec, 2008). Further, the maturity principle in personality psychology suggests that an age related increasement in the traits of agreeableness, conscientiousness, in addition to emotional stability, could take place (Caspi et al., 2005). Regarding military contexts, a longitudinal German study found that training in conventional forces was associated with a lasting reduction in agreeableness, as measured 5 years after the end of service (Jackson et al., 2012). This finding demonstrates a possible impact of military experience on agreeableness, opposite the change direction forecasted by the general maturity principle.

Published empirical personality studies of employees in SOFs are scant, most likely due to security issues and the secretive culture typically surrounding such units. Two Norwegian studies, using applicants, investigated the predictive validity of a Big Five test used in the operator selection to the MJK, but findings did not reveal clear associations between test scores and pass/fail results in an upcoming strenuous practical selection period (Hartmann et al., 2003; Hartmann and Grønnerød, 2009). The authors of the 2003 study did claim, however, that high emotional stability and low extraversion increased the probability of success. Bartone et al. (2008) noted that prediction of success in selection courses for elite military units have been met with limited success. These authors did, however, find a significant group difference in mean scores between those who passed and those who failed a 4-week selection and assessment course among United States Army Special Forces candidates, when investigating a measure of psychological hardiness. The authors described hardiness as a personal stress-resiliency resource and argued for the relevance of this psychological construct in SOFs.

A study of 139 NEO-PI-R profiles from operators in the United States Navy Sea-Air-Land (SEAL) Commando, found that SEALs had lower scores on neuroticism and agreeableness, the same to lower on openness, and higher scores on conscientiousness and extraversion, relative to the norms for adult American males (Braun et al., 1994). This study also reported that more-experienced SEALs scored higher on conscientiousness and lower on extraversion than less-experienced SEALs, and that commissioned officers had higher scores on both of these factors when compared to enlisted operators. Regarding the differences in personality trait levels based on amount of experience, the authors concluded that age was responsible for the effect, not warfare experience *per se*. A more recent study of a police Special Force reported that its personnel had significantly higher scores on all five factors (emotional stability being reversed neuroticism) when compared to the population mean scores of males, and also for career soldiers, in Italy (Garbarino et al., 2012). Police Special Forces can be somewhat different from military SOFs in their primary mission forms, although obvious similarities exist regarding stringent personnel selection, advanced tactical training, and special missions such as counter-terrorism (Johnsen, 2017).

Although not investigating Big Five traits *per se*, a recent Norwegian study by Boe et al. (2017) investigating character strengths in the FSK sheds light on desired personality functioning in this unit. The authors reported that 27 officers from the FSK evaluated the character strength named open-mindedness as the most important for successful duty as a Special Forces officer, and that this sample evaluated humility/modesty, curiosity, and forgiveness and mercy as more important compared to character strength evaluations done by Norwegian Army officers.

At a more general level for “high-risk operational personnel,” such as SOF-operators, clandestine intelligence operatives and astronauts, Picano and Roland (2012) wrote that six attribute dimensions are commonly required for successful performance: emotional stability, adaptability, teamwork abilities, physical stamina and fitness, sound judgment and decision-making, and intrinsic motivation. The first three of these attribute dimensions point to a low degree of neuroticism, and shed light on the relevance of the interpersonal traits of extraversion and agreeableness. Intrinsic motivation can perhaps be related to the conscientiousness factor, a trait that has demonstrated predictive validity across different jobs in both civilian and military settings (Barrick and Mount, 1991; Salgado, 1998; Darr, 2009).

Summarized, the personality picture in SOFs, whether it is for applicants or employees, or for the broader category of high-risk operational personnel, is not necessarily a clear one, except for repeated findings of low neuroticism (high emotional stability). The lack of success in predicting performance in SOF selections based on personality variables, and the scant empirical personality findings of employees, clouds the picture. By reporting on additional personality data, the present study contributes further to the personality psychology knowledge base of SOFs.

### Aims of the Study

The purpose of this study was to investigate personality characteristics of personnel in the NORSOF by using a Big Five test called the Norwegian Military Personality Inventory (NMPI). NMPI-data were obtained from three personnel categories, including: (1) FSK-operator, (2) MJK-operator, and (3) SOF-support. We also had information on age, number of combat-deployments and whether the participants were educated as officers (OF) or not (OR – other ranks). By investigating the impact of personnel category, age, number of combat-deployments and rank on levels of personality traits, a discussion of the organizational psychology within the NORSOF can be done – more specifically, we hypothesized that if clear personality dissimilarities were found, this could be a challenge for the climate among colleagues. Further, a database consisting of NMPI scores of applicants to basic officer training (1-year education to become a non-commissioned officer – NCO) in the Norwegian Armed Forces was used for comparative analyses. Such analyses are relevant for examining whether the SOF-operators have a different personality profile than other categories of military personnel, considering the differences in selection systems and subsequent military service for SOFs and conventional forces, respectively. We set forth to investigate if the “specialness” of the operators was reflected in their personalities. Three research questions (RQ) were formulated for this study:

(1) For the total NORSOF sample, are there significant group-differences in personality traits based on age groups, number of combat-deployments groups, and rank (OF/OR)?
(2) Are there significant group-differences in personality traits between the three NORSOF personnel categories (FSK-operator, MJK-operator, and SOF-support)?
(3) Are there significant group-differences in personality traits between SOF-operators (who were all males) and male NCO-applicants?

Based on the maturity principle in personality psychology (Caspi et al., 2005), we expected that older NORSOF personnel would score higher on agreeableness, conscientiousness and emotional stability than younger colleagues. We were unsure of potential differences based on combat-deployments and rank, however, especially since these variables were not strongly associated with age in our sample. Considering the SOF status of both the FSK and the MJK, we had no educated reason to expect clear differences in levels of personality traits between operators in the two units, even though these Special Forces divisions are separate and have their historical roots in the Army and Navy, respectively. Based on previous studies (Braun et al., 1994; Bartone et al., 2008; Garbarino et al., 2012), we expected that SOF-operators would score higher on emotional stability and conscientiousness than SOF-supports and NCO-applicants. Serving as one of “the quiet professionals,” a term often used to describe Special Forces operatives, a lower degree of extraversion and agreeableness were also expected – although higher extraversion relative to the population was found in the studies of Braun et al. (1994) and Garbarino et al. (2012). The study of Jackson et al. (2012), demonstrating that military training was associated with agreeableness reduction, further supported this expectation, although we did not find personality change studies from SOF environments. The study of Boe et al. (2017) gave expectations of higher openness to experience among operators relative to other personnel categories.

## Materials and Methods

### Participants

Personnel employed by the NORSOF were asked to participate in this study. Conscripts in the “Fallskjermjegertroppen” (Paratroopers) and the all-female “Jegertroppen” (Hunter Troop) were not included, as these soldiers are not employees. The final sample was *N* = 190. The response rate is unknown, as the actual size of this military department is classified information. Personnel selected and trained as SOF-operators either in the FSK or the MJK constituted 76 individuals (40%) in the final sample, whereas the remaining 113 (60%) were support personnel (one person did not report background).

### Measures

#### Demographic Information

The questionnaire used included the following demographic variables: Background (FSK-operator; MJK-operator; other); Age (under 30; 30–40; above 40); Number of deployments (0; 1–5; 6–10; above 10); Rank (Other Ranks; Officer Rank; Civilian). Age and number of deployments were coded in categories for minimizing anonymity concerns of respondents. A deployment in the NORSOF means participation in international combat operations, in which the duration can vary. In the last decade, personnel in the NORSOF have usually been deployed 4–6 months at a time in international conflict areas. To gain an officer rank in Norway, a 3-year-long education at a military academy either in the Army, Navy or Air Force, resulting in a Bachelor’s degree, is required.

#### Norwegian Military Personality Inventory (NMPI)

The Big Five personality dimensions were measured using the NMPI. This is a self-report seven-point Likert scale factor-level test consisting of 79 items, developed by the Norwegian Armed Forces (Antonsen, 2016; Skoglund, 2017; Skoglund et al., unpublished). The five factors (number of items in parenthesis) of the NMPI are called Extraversion (18), Agreeableness (16), Conscientiousness (13), Emotional Stability (15), and Openness to new experiences (17). Skoglund et al., (unpublished) reported Cronbach’s alphas of α = 0.78–0.90 for the NMPI factors, and the following correlations between the NMPI factors and the NEO-PI-3 factors based on a sample of 850 applicants for basic officer training (NCO): E, *r* = 0.80; A, *r* = 0.62; C, *r* = 0.82; ES (reversed Neuroticism), *r* = −0.82; O, *r* = 0.80. For the sample in the present study, Cronbach’s alphas for the NMPI factors were: α = 0.88 for E and A; α = 0.85 for C; α = 0.87 for ES; α = 0.75 for O.

After promising initial validation findings (Antonsen, 2016; Skoglund, 2017), the NMPI are undergoing a research phase in the NAF, where norms and practical usage aspects are yet to be developed (at the time of writing). The authors of the present study, therefore, had limited choices regarding comparative analyses between the SOF-sample and other samples, and thus formulated RQ3 in line with accessible datasets. NMPI’s short form, in-house copyright permission and complete data control without third parties were necessary requirements set forth by the NORSOF for this study. Thus, we could not use established tests, such as the NEO-PI-3 (McCrae and Costa, 2010).

### Procedure

The Norwegian Social Science Data Service, the research commission at the Norwegian Defence University College, and the NORSOF approved this study. The Chief Psychologist in the Norwegian Armed Forces has the copyright permission for the NMPI, and gave permission to use the test for research purposes. The questionnaire with demographic variables and the NMPI were distributed through a military mail-system during May 2018.

### Statistical Analyses

IBM SPSS 25.0 was used for all statistical analyses. Descriptive analyses investigated characteristics of the sample, whereas one-way between-groups analyses of variances (ANOVAs) and independent-samples *t*-tests were conducted for comparing groups. Missing values were excluded analysis by analysis. Effect sizes are reported as eta squared for ANOVAs and as Cohen’s *d* for *t*-tests. Eta squared and Cohen’s *d* were interpreted in line with Cohen (1988), classifying 0.01 and 0.2 as a small effect, 0.06 and 0.5 as a medium effect and 0.14 and 0.8 as a large effect, respectively.

## Results

### Demographic Information and Evaluation of Parametric Assumptions

Table 1 presents a summary of demographic information for the sample. There were more participants reporting a background as an FSK-operator compared to an MJK-operator. All participants reporting operator backgrounds were males. Having asked for background in the questionnaire, the authors did not have information of the proportion of operators no longer on active duty as operatives in saber squadrons (operational units). There were a few females in the SOF-support group, but not enough for analyses of potential gender differences in personality scores. Age distribution had a somewhat similar shape for the FSK-operators and the MJK-operators, whereas there were a higher proportion of participants above 40 years of age in the larger SOF-support group. The majority of participants in all three groups had one to five combat-deployments. The majority of both operator groups had a specialist rank (OR), whereas there were a greater proportion of participants with officer ranks (OF) in the support group. As only a handful reported civilian positions, they were removed from the sample. There was one missing value on the variables of rank, background and number of combat-deployments.

**TABLE 1:** Demographic information of the sample (in percentages).

Demographic variable	FSK-operators (*N* = 48)	MJK-operators (*N* = 28)	SOF-supports (*N* = 113)
**Gender**			
Male	100	100	92.9
Female	–	–	7.1
**Age**			
<30	20.8	14.3	23.9
30–40	54.2	57.1	36.3
>40	25.0	28.6	39.8
**Number of deployments**			
None	4.2	3.6	13.4
1–5	60.4	78.6	66.1
6–10	35.4	17.9	17.9
10	–	–	2.7
**Rank**			
Officer (OF)	33.3	39.3	56.6
Other (OR)	66.7	60.7	43.4

Investigating the total NORSOF sample, the score distributions on the NMPI factors of agreeableness, conscientiousness and emotional stability were somewhat negatively skewed (−0.95, −0.47, and −0.56, respectively), whereas the distribution of scores on extraversion and openness were quite symmetrical (0.04 and −0.23, respectively). A visual inspection of histograms and Q-Q (quantile-quantile) plots revealed no serious violations of normality, legitimizing usage of parametric testing. When extracting the SOF-operators as a group, the same pattern in score distributions emerged. Further, Levene’s test for homogeneity of variance are reported on for the specific statistical tests used.

### Research Question 1

One-way between-groups ANOVAs and independent samples *t*-tests were conducted to explore the impact of age, number of combat-deployments and rank on NMPI factor scores. The separate analyses of age and number of combat-deployments were supported by a correlation of *r* = 0.39 between the two variables, indicating a relationship of a low-medium magnitude (Cohen, 1988). To gain statistical power (that is, avoiding too few participants in the demographics categories), the total NORSOF sample was used for analyses investigating RQ1. Levene’s tests for homogeneity of variance were not significant for the analyses related to RQ1, with the exception of agreeableness scores based on the grouping variable of combat-deployments (*p* < 0.05). As this indicated a violation of homogeneity of variance, Welch’s *F* test was used for this specific analysis. Precisions of statistically significant findings are indicated by the 95% confidence intervals of the differences between the means.

Table 2 summarizes the ANOVA analyses. There was a statistically significant difference in emotional stability scores for the three age groups (under 30, 30–40, above 40): *F*(2,187) = 5.17, *p* = 0.007. The eta squared effect size was 0.05. *Post hoc* comparisons using the Tukey HSD test demonstrated that the mean score for those less than 30 years of age (*M* = 5.16, *SD* = 0.73) was significantly different from those above 40 years of age (*M* = 5.63, *SD* = 0.72), 95% CI [0.09, 0.58].

**TABLE 2:** NMPI factor means, standard deviations and one-way between-groups analyses of variance using age, number of combat-deployments and personnel category as predictors.

	Age				
	
NMPI factor	Group 1 (*N* = 41): under 30	Group 2 (*N* = 83): 30–40	Group 3 (*N* = 66): above 40	*F* (η^2^)	Significant effects
Extraversion	4.53 (0.88)	4.74 (0.79)	4.85 (0.82)	1.95 (0.02)	ns
Agreeableness	5.43 (0.70)	5.28 (0.69)	5.37 (0.79)	0.70 (0.01)	ns
Conscientiousness	5.74 (0.74)	5.65 (0.71)	5.52 (0.68)	1.31 (0.01)	ns
Emotional stability	5.16 (0.73)	5.40 (0.77)	5.63 (0.72)	5.17* (0.05)	1 < 3
Openness	4.74 (0.65)	4.84 (0.53)	4.93 (0.59)	1.38 (0.01)	ns

	**Number of combat-deployments**				
	
	**Group 1 (*N* = 18): none**	**Group 2 (*N* = 126): 1–5**	**Group 3 (*N* = 45): above 5**		

Extraversion	4.92 (0.72)	4.66 (0.81)	4.87 (0.85)	1.61 (0.02)	ns
Agreeableness^1^	5.81 (0.61)	5.32 (0.63)	5.24 (0.94)	4.34* (0.04)	1 > 2, 1 > 3
Conscientiousness	5.92 (0.66)	5.62 (0.73)	5.54 (0.67)	1.88 (0.02)	ns
Emotional stability	5.15 (0.70)	5.40 (0.75)	5.64 (0.75)	3.22* (0.03)	1 > 3
Openness	4.96 (0.61)	4.82 (0.57)	4.93 (0.56)	0.89 (0.01)	ns

	**Personnel category**				
	
	**Group 1 (*N* = 48): FSK-operator**	**Group 2 (*N* = 28): MJK-operator**	**Group 3 (*N* = 113): SOF-support**		

Extraversion	4.61 (0.74)	4.75 (0.97)	4.78 (0.82)	0.66 (0.01)	ns
Agreeableness	5.31 (0.63)	5.17 (0.77)	5.40 (0.75)	1.19 (0.01)	ns
Conscientiousness	5.68 (0.75)	5.61 (0.68)	5.61 (0.70)	0.17 (0.00)	ns
Emotional stability	5.55 (0.78)	5.51 (0.80)	5.35 (0.74)	1.38 (0.01)	ns
Openness	4.86 (0.51)	4.88 (0.50)	4.84 (0.63)	0.05 (0.00)	ns

^1^Welch’s *F* and Games-Howell *post hoc* comparison used; *p < 0.05; η^2^, eta squared; < or >, significant group differences after *post hoc* comparisons using the Tukey HSD test; ns, non-significant.

There were only three participants who had more than 10 combat-deployments, thus the categories for number of combat-deployments were re-coded into three groups (None; 1–5 deployments; above five deployments). There was a statistically significant difference in scores on agreeableness for the three groups: *Welch’s F*(2,41.61) = 5.33, *p* = 0.009. The eta squared effect size was 0.04. *Post hoc* testing using the Games-Howell procedure showed that the mean score of those without any deployments (*M* = 5.81, *SD* = 0.61) was significantly different from those with both 1–5 deployments (*M* = 5.32, *SD* = 0.63), 95% CI [0.06, 0.91], and those with more than five deployments (*M* = 5.24, *SD* = 0.94), 95% CI [0.10, 1.04].

In addition, there was a statistically significant difference in scores on emotional stability for the three groups *F*(2,186) = 3.22, *p* = 0.042. The eta squared effect size was 0.03. *Post hoc* comparisons using the Tukey HSD test demonstrated that the mean score for those without any deployments (*M* = 5.15, *SD* = 0.70) was significantly different from those with more than five deployments (*M* = 5.64, *SD* = 0.75), 95% CI [0.01, 0.98]. These statistically significant differences in agreeableness and emotional stability based on combat-deployments are, however, somewhat imprecise (large confidence intervals) – most likely due to the small sample size in the non-deployment group (*N* = 18).

Finally, for RQ1, independent-samples *t*-tests were conducted to explore differences in mean factor scores based on rank, OR (*N* = 98) and OF (*N* = 91). There was a statistically significant difference in extraversion scores for the OR group (*M* = 4.54, *SD* = 0.79) and the OF group [*M* = 4.93, *SD* = 0.81; *t*(187) = −3.41, *p* = 0.00, two-tailed], 95% CI [0.17–0.63]. The effect size for this difference, using Cohen’s *d*, was 0.50.

### Research Question 2

The lower part of Table 2 presents the NMPI mean factor scores of FSK-operators, MJK-operators, and the SOF-support group, and summarizes results from ANOVAs relevant for RQ2. The Levene’s tests for homogeneity of variance were not significant for the analyses related to RQ2, thus indicating equal variances. A visual inspection revealed quite similar mean factor scores for the three groups. One-way between-groups ANOVAs did not show any statistically significant differences in NMPI factor scores between the three groups, although it should be noted that the sample sizes of FSK- and MJK-operators are quite small with 48 and 28 individuals, respectively. A power analysis using the G^∗^power software (Faul et al., 2009) showed that the projected sample needed for a detection of a mean score difference between two groups with an effect size of Cohen’s *d* = 0.50, and with alpha = 0.05 and power = 0.80, is *N* = 64 in each group. Thus, FSK- and MJK-operators were categorized as one group containing 76 individuals. Independent-samples *t*-tests were conducted to investigate differences in NMPI-factor mean scores between the SOF-operator group and SOF-supports, resulting in no significant differences for any of the five factors.

### Research Question 3

This study had access to a database used for validation purposes of the NMPI, based on scores from applicants attending a common assessment center for basic officer training (NCO) in the Army, Navy and Air Force in the summer of 2016 (Antonsen, 2016; Skoglund, 2017; Skoglund et al., unpublished). All participants at the assessment center were asked to participate in the validation study. They were informed that the NMPI was attached to a research project, and that scores on this test would not affect upcoming selection decisions. The final sample of NCO-applicants with NMPI factor scores were *N* = 843–856, giving a response rate above 90%. As all NORSOF-operators were males, male NCO-applicants were used for answering RQ3, controlling for possible gender-effects. Because of missing data on some items, there were 648–662 males with NMPI factor scores in the sample – this was approximately 77% of the total NCO-sample. Among the males, age varied from 18 to 34 (*M* = 19.62, *SD* = 1.89). The applicants had limited military experience (about half did not have any) before participating in the assessment center, in which 53% of the males either canceled the selection phase themselves or were evaluated too low on the selection criteria.

Independent-samples *t*-tests were conducted to investigate RQ3. Equal variances for the two groups were confirmed by non-significant results on Levene’s tests. As Table 3 shows, there were three findings reaching statistical significance.

**TABLE 3:** NMPI mean factor scores, standard deviations and independent samples *t*-tests for SOF-operators and male NCO-applicants.

	SOF-operators (*N* = 76)		NCO-applicants (*N* = 648–662)			
		
NMPI factor	*M*	*SD*	*M*	*SD*	*t*	Cohen’s *d*
Extraversion	4.67	0.83	5.16	0.76	−5.37*	0.62
Agreeableness	5.26	0.68	5.65	0.68	−4.72*	0.57
Conscientiousness	5.65	0.72	5.65	0.69	0.08	0.00
Emotional stability	5.54	0.78	5.33	0.82	2.07*	0.26
Openness	4.87	0.50	4.83	0.61	0.59	0.09

*p < 0.05.

A statistically significant difference in mean scores on extraversion was found for the SOF-operator group (*M* = 4.67, *SD* = 0.83) and NCO-applicant group [*M* = 5.16, *SD* = 0.76; *t*(722) = −5.37, *p* = 0.00, two tailed], 95% CI [0.32, 0.68]. For mean scores on agreeableness, there was also a statistically significant difference between SOF-operators (*M* = 5.26, *SD* = 0.68) and NCO-applicants [*M* = 5.65, *SD* = 0.68; *t*(736) = −4.72, *p* = 0.00, two tailed], 95% CI [0.23, 0.55]. Finally, there was a significant difference in mean scores on emotional stability for SOF-operators (*M* = 5.54, *SD* = 0.78) and NCO-applicants [*M* = 5.33, *SD* = 0.82, *t*(729) = 2.07, *p* = 0.04, two tailed], 95% CI [0.01, 0.40]. Cohen’s *d* effect sizes were 0.62 for extraversion, 0.57 for agreeableness, and 0.26 for emotional stability.

## Discussion

The present study is the first to report on Big Five personality traits of Norwegian SOF personnel, by using the NMPI. The NMPI is a factor-level test demonstrating sound psychometric properties, and strong convergent validity toward the NEO-PI-3 (Antonsen, 2016; Skoglund et al., unpublished). Three research questions investigating the impact of demographic information and different personnel categories on levels of personality traits were formulated. Of special interest were potential personality differences between SOF-operators and other personnel categories, considering the uniqueness in the selection, training and mission forms of the former compared to the latter (Spulak, 2007; NATO, 2012; Berg-Knutsen and Roberts, 2015). Statistically significant findings were found. Care in the interpretation of results is warranted, though, primarily since the actual mean differences observed in scores on personality traits were small. Results demonstrated firstly that younger personnel in the NORSOF were somewhat lower on emotional stability than their older colleagues, and that those without any combat-deployments scored a little higher on agreeableness and slightly lower on emotional stability relative to employees with such experiences. In addition, personnel with officer ranks (OF) reported, to some degree, higher extraversion compared to specialists (OR). Secondly, there were no significant differences in personality trait scores between SOF-operators from both the FSK and the MJK, and the SOF-support group. Operators from the two units had very similar mean personality trait scores (a mean difference of 0.08 for the five factors), and there were no significant differences in mean trait scores between SOF-operators as a group and SOF-support personnel. Thirdly, compared to applicants for basic officer training (to become an NCO) in the conventional forces, SOF-operators were less extroverted, less agreeable and to a certain extent more emotionally stable.

The results demonstrate an impact tendency of age and number of combat-deployments on emotional stability levels. This points to possible important organizational psychological characteristics within the NORSOF. As older operators and support personnel, as well as those with combat-deployment experiences, reported higher scores on emotional stability relative to younger and less-experienced ones, they can be well-suited role models in a military department where managing stressors is of great importance. Contrary to our findings, Braun et al. (1994) did not, however, report differences in neuroticism based on age or warfare experience among SEALs. The finding regarding age is in line, though, with general research on personality trait change in adulthood, where a decrease in neuroticism is sometimes seen (Caspi et al., 2005; Roberts and Mroczec, 2008).

While it may be obvious that high levels of neuroticism can be counterproductive in stressful settings, high levels of agreeableness have somewhat contradictorily been associated both with less stressors in life (Leger et al., 2016), and with an increase in subjective distress when encountering interpersonal conflicts (Suls et al., 1998). Thus, one can argue that different levels of agreeableness seem both dysfunctional and functional, perhaps depending on the levels of other traits and on contextual factors. It may be that combat-deployments, thus seeing conflict and war up close, can take its toll on the characteristics associated with agreeableness – although causality cannot be inferred from the present study. Trust, altruism and tender-mindedness, the NEO facets correlating most strongly to the NMPI factor agreeableness (Skoglund et al., unpublished), are necessary building blocks for team cohesion, but can be exploited if such tendencies are too strong toward others who have hostile intentions. Those with combat-deployment experiences may have “balanced” their agreeableness more than those who have not been deployed yet, or maybe the more experienced personnel have this balance naturally. Braun et al. (1994) did not, however, find any differences in agreeableness based on amount of experience of SEALs, but noted that personality differences based on warfare experience were most likely explained by age. Except for the difference in scores on emotional stability based on age groups, we did not obtain results in line with expectations based on the maturity principle in personality psychology (Caspi et al., 2005). The differences in agreeableness based on number of combat-deployment are somewhat in line, though, with the findings demonstrating that conventional military training can reduce levels of this trait (Jackson et al., 2012).

The finding that officers (OF) reported themselves as slightly more extraverted relative to specialists (OR), gives rise to speculations of a possible functionality of this trait in the selection to the 3-year-long officer education in Norway and the later performance of leadership. More often than not, operators and support personnel in the NORSOF attend the officer education after some years of service as specialists, pointing to a possibility that the extraverted are more likely to become officers, or that the less extraverted prefer to be specialists. This finding is in line with results from Braun et al. (1994), as these authors reported higher degrees of extraversion among SEAL-officers relative to SEAL-enlisted personnel. The comparison to the SEALs, however, is somewhat imprecise as our sample were operators and support personnel combined.

The empirical studies of Bartone et al. (2008) and Garbarino et al. (2012) support the theory of “specialness” of SOFs by demonstrating differences in psychological hardiness and Big Five scores among successful and non-successful SOF-applicants and police Special Force officers and career soldiers, respectively. Comparing operators to population norms, these studies, together with the Braun et al. (1994) study, reported significant differences in Big Five trait levels, further underlining a kind of specialness. The operators of the FSK and the MJK were similar in their Big Five personalities, and they did not differ significantly from support personnel. Thus, there were no clear intradepartmental differences regarding personality trait levels, suggesting that the specialness of SOF-operators was not reflected in their personalities when compared with personnel within the NORSOF.

The present study did, however, support the theory of specialness when comparing SOF-operators with NCO-applicants. A personality profile of the operator emerged, characterized by lower extraversion and agreeableness, and somewhat higher emotional stability, relative to male applicants to basic officer training in conventional forces. The effect sizes in Cohen’s *d* were medium for differences in extraversion and agreeableness, and small for the difference in emotional stability. Although not directly comparable to the male population in Norway, these applicants were not militarily experienced individuals, and about half of them did not manage successful NCO-selection. Both SEALs (Braun et al., 1994) and Special Forces police officers (Garbarino et al., 2012) had higher scores on extraversion relative to population norms, and also when compared to career-soldiers for the police operators, which can be said to be opposite to our findings (although we did not compare to population norms). Our finding of lower agreeableness is in line with Braun et al. (1994), but not with Garbarino et al. (2012). Emotional stability has especially been highlighted as important for high-risk operational personnel (Braun et al., 1994; Bartone et al., 2008; Garbarino et al., 2012; Picano and Roland, 2012), and it may not come as a surprise that the present study found the same trend as earlier empirical studies, demonstrating higher levels of this trait for SOF-operators relative to other samples. The SOF personality profile that emerged is somewhat contradictory on a conceptual level to the character strengths study of Boe et al. (2017), where open-mindedness was evaluated as especially important by FSK-officers, and where humility/modesty, curiosity, and forgiveness and mercy were evaluated as more important by these officers, relative to evaluations done by officers in the conventional Army. It could be that Big Five personality traits and character strengths tap into different psychological phenomena, although this is debated (Peterson and Seligman, 2004).

Braun et al. (1994) suggested a personality description of the “average” SOF-operator (SEAL) based on their findings, resulting in a profile that is intuitively comparable to what one may think is functional for high-risk operational personnel, highlighting such attributes as hardiness and persistence, and some skepticism of others’ intentions. Where these authors had access to population norms of the personality test used (the NEO), we did not. Nonetheless, interpreting the findings in the present study, it becomes clear that the Norwegian “tip of the spear” operators typically do not have very low or very high scores on any of the Big Five factors, and that they tend to be less extraverted and agreeable, and more emotionally stable, when compared to those who seek general purpose forces. This profile is not counter-intuitive, and implies an overall flexible personality functioning, drawing a picture of an emotionally stable individual with an initial reserved attitude toward strangers. Considering the future SOF-operator termed the “Warrior-Diplomat” (Berg-Knutsen and Roberts, 2015), operating in diverse contexts, we suggest that this profile is adaptive. Being emotionally stable and somewhat cautious with interpersonal interactions may be seen as functional for serving in a unit operating in high-stress environments, and in which security issues and secrecy are necessary. Raw scores on the interpersonal traits of extraversion and agreeableness were not low, making it reasonable to think of the SOF-operator as socially adept if the circumstances call for diplomatic attitudes.

As the emerged average personality profile of the operator is interpreted as functional, the selection processes in both the FSK and the MJK seem to function well regarding the evaluation of personality characteristics of applicants. The personality tests implemented in the NORSOF-operator selections serve primarily as background materials for military psychologists’ advices concerning applicants’ strengths and weaknesses. This practice is comparable to the procedure in the Norwegian Police Special Operations Officer selection, as documented by Johnsen (2017). For an optimal evaluation of personality test use, both the possibility of personality change based on military experience (Jackson et al., 2012) and the perspective of trait activation based on situational cues (Tett and Burnett, 2003; Judge and Zapata, 2015) could be important aspects to consider. The first point could be researched upon with a repeated measures design. The authors suppose, though, that it would be challenging to operationalize a sound situational taxonomy matching SOF job performance, in which a persistent adaptability to unforeseen circumstances, in high stress environments, is the primary attribute to select for. Personality factors associated with this attribute in a selection context are perhaps not obvious, with the exception of broadly formulated characteristics (Picano and Roland, 2012).

Study limitations should be noted. Sampling bias is an unknown factor in this study, due to the confidentiality of the number of employees at the time of data extraction. We do not know the response rate and the exact representativeness of study-participants for the NORSOF as a whole, although we note that conscript personnel were not included in this study. Our final sample size was small, primarily because NORSOF employees are few in numbers and belong to one of the most secretive branches in our society. The results from the analyses, therefore, require cautiousness in interpretation. Our sample is nonetheless valuable for the military psychology literature, considering the scant empirical studies from such highly specialized environments. The personality measure used, the NMPI, is a newly developed Big Five measure for the Norwegian Armed Forces. Although validated with good results, norms are yet to be developed, and operational use of the test in the military has not commenced at the time of writing. Specifically, an unfortunate limitation was the missing possibility of comparative analyses between the NORSOF sample and population norms. Of special note for the present study is the difference in age for the NORSOF sample and the NCO-applicants; ideally, personality comparative data should be on the same age groups, considering possible changes in some personality traits with increased age. The reported personality trait differences would perhaps be more salient, resulting in larger effect sizes, if comparative analyses were done with civilian samples, and population norms. Some range restriction in the personality scores of NCO-applicants is reasonable to assume, as this group was preselected through the conscript assessment procedures in Norway. Demand characteristics could also be relevant for the NMPI scores of NCO-applicants, as the data-collection for this sample was done in a selection context.

## Conclusion

In conclusion, the findings demonstrated that FSK-operators, MJK-operators and SOF-supports did not differ in a clear way in their group personality trait scores. Therefore, the SOF environment in Norway seems to attract and recruit people that have similarities in their Big Five makeup, although there are different selection systems for the operators and diverse military backgrounds among the supports. The authors did not find this counterintuitive, as being employed by the NORSOF is only for the few – although our expectations of differences between the operators and the supports were not met. The climate among colleagues is of course dependent on many factors, one being personality variables. We did not find clear personality-based obstacles for cooperation between the departments. Questions related to potential organizational benefits with increased personality diversity in the NORSOF are unanswered based on the present study. This study replicated the most consistent findings regarding the personalities of high-risk operational personnel, namely higher emotional stability among SOF-operators compared to other samples. This finding is, however, somewhat questionable regarding the small effect size (*d* = 0.26), and the age difference between the operators and the NCO-applicants (most operators were in their 30s, whereas the applicants had a mean age of 19–20). More contradictory findings relative to earlier studies are the results of lower agreeableness and extraversion among SOF-operators compared to the NCO-applicants, although this was in line with our expectations. It was surprising, however, that the operators did not score higher than NCO-applicants on conscientiousness – especially since this trait has demonstrated predictive validity for job performance in military settings (Salgado, 1998; Darr, 2009). The expectation of higher openness was not met, perhaps due to construct differences in character strengths and Big Five personality traits. Nonetheless, this study supports that these carefully selected men are somewhat special as aspects of their personalities are a little bit different compared to those who apply for service in general purpose forces. The authors also note that the ability of the NMPI to detect differences between military personnel categories further supports the validity of this test.

Future quantitative studies concerning psychological characteristics in SOF environments should be aware of sampling bias and should acknowledge the importance of statistical power, while balancing this with the fact that samples from these secretive environments can be challenging to obtain. Where possible, studies should compare the personalities of SOF personnel with civilian samples for further investigation of the assumed uniqueness of the operators. Repeated personality measures would be valuable for investigating the possible impact of the SOF experience on personality traits. Based on findings from the present study, controlling for the effect of age, number of combat-deployments and rank can be important for investigating personality questions in SOFs. We suggest that the Big Five personality profile that emerged for the average NORSOF-operator is a functional one considering the Warrior-Diplomat role required in modern Special Forces operations, and that some cautiousness in interpersonal settings is functional when serving as one of “the quiet professionals.”

## Data Availability Statement

The data analyzed in this study were obtained from the Norwegian Special Operations Command and the Norwegian Defence University College. Due to a confidentiality clause, the raw data will not be made publicly available. Requests to access these datasets should be directed to the Norwegian Defence University College (mail: post.fhs@mil.no).

## Ethics Statement

The studies involving human participants were reviewed and approved by The Norwegian Social Science Data Service. The patients/participants provided their written informed consent to participate in this study.

## Author Contributions

TS wrote the manuscript. All authors analyzed and interpreted the data and gave feedback on the manuscript. T-HB designed the study and collected the data.

## Conflict of Interest

The authors declare that the research was conducted in the absence of any commercial or financial relationships that could be construed as a potential conflict of interest.
